# Thermodynamic Properties of γ- and δ-Lactones: Exploring Alkyl Chain Length Effect and Ring-Opening Reactions for Green Chemistry Applications

**DOI:** 10.3390/molecules30020399

**Published:** 2025-01-18

**Authors:** Ana L. R. Silva, Gastón P. León, Vladimír Lukeš, Erik Klein, Maria D. M. C. Ribeiro da Silva

**Affiliations:** 1 Centro de Investigação em Química (CIQUP), Institute of Molecular Sciences (IMS), Department of Chemistry and Biochemistry, Faculty of Sciences, University of Porto, Rua do Campo Alegre, 4169-007 Porto, Portugal; gaston.leon@fc.up.pt (G.P.L.); mdsilva@fc.up.pt (M.D.M.C.R.d.S.); 2 Institute of Physical Chemistry and Chemical Physics, Slovak University of Technology in Bratislava, Radlinského 9, 812 37 Bratislava, Slovakia; vladimir.lukes@stuba.sk

**Keywords:** γ-undecanolactone, δ-undecanolactone, enthalpy of formation, enthalpy of vaporization, combustion calorimetry, Calvet microcalorimetry, quantum chemical calculations

## Abstract

An extensive thermochemical study of γ-undecanolactone and δ-undecanolactone has been developed using two complementary calorimetric techniques. The combustion energy of each compound was determined by static-bomb combustion calorimetry, and the corresponding enthalpy of vaporization was determined by high-temperature Calvet microcalorimetry, in which both properties of each compound are reported at *T* = 298.15 K. The standard molar enthalpy of formation in the gas phase of each lactone was derived by the combination of the experimental results. Additionally, high-level computational calculations were carried out, using composite ab initio G4 and G4(MP2) methods, as well as DFT M06-2X/6-311++G(d,p) approach, to estimate the corresponding enthalpy of formation in the gas phase. The experimental and computational results are in good agreement. The G4 and G4(MP2) methods show the best accordance with experimentally determined gas phase enthalpies of formation. The experimental results are discussed in terms of structural contributions to the energetic properties of the lactones studied, as well as to other alkylated γ- and δ-lactones, and empirical correlations are suggested for the estimation of the standard molar enthalpies of formation, at *T* = 298.15 K, for other alkylated γ- and δ-lactones, both in the liquid and gaseous phases, as well as for the respective enthalpies of vaporization. Finally, the thermochemistry of individual steps of lactone ring opening and successive decarboxylation mechanism, including the identification of transition states, was studied using the M06-2X/6-311++G(d,p) approach.

## 1. Introduction

Lactones are cyclic carboxylic esters derived from hydroxy carboxylic acids. The γ-lactones and the δ-lactones with 5- and 6-membered rings, respectively, represent the most diverse, chemically stable groups of naturally occurring lactones. They are present in fruits, vegetables, meat, milk, butter, cheese, and wine and substantially contribute to their aroma and flavor. Apart from their applications in food and cosmetic industries, γ- and δ-lactones can be employed as non-toxic solvents, as well as utilized in the production of bio-degradable polymers, or as fuels [1,2]. Additionally, lactones show a variety of biological activities, such as antimicrobial, anti-inflammatory, or antitumor [3].

The availability of data related to energetic properties of these compounds has primordial relevance for the characterization of their role in the processes where they are involved. Also, the insights into the effect of the alkyl chain length on their energetic properties offer valuable data for various processes involving lactones in chemistry and biological systems, namely in the valorization of biomass-derived intermediates [4,5]. Despite this, reports on most of these properties are scarce in the literature, having already led us to develop an experimental and computational thermochemical study of some biomass-derived cyclopentenones and lactones [6,7]. The experimental and theoretical works available in the literature are focused on particular chemical reactions of lactones and formed products.

**Figure 1:**
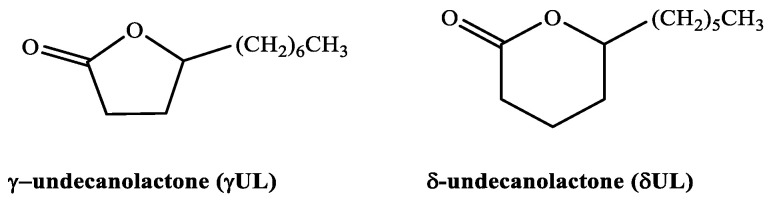
Structural formulae of γ-undecanolactone and δ-undecanolactone.

Concerning these facts, in this work, we present a systematic experimental and theoretical study on the thermodynamic properties of two suitable model lactones for biomass valorization, γ-undecanolactone, γUL (IUPAC name: 5-heptyloxolan-2-one) and δ-undecanolactone, δUL (IUPAC name: 6-hexyloxan-2-one), whose structures are shown in Figure 1. The standard molar enthalpy of combustion in the liquid phase and the standard molar enthalpy of vaporization of each compound are determined calorimetrically: the combustion experiments are performed by static-bomb combustion calorimetry and the vaporization study is performed using a high-temperature Calvet microcalorimeter. Therefore, the standard molar enthalpy of formation in the gas phase, at 298.15 K, has been evaluated based on the experimental data obtained from the study of the compounds. In addition to this, we intended to study the correlation energy vs. the structure of the lactones studied, and other alkylated δ-and γ-lactones which are already available in the literature. The second aim of this work is to calculate the enthalpies of formation of the two lactones using the composite ab initio G4 and G4(MP2) methods, as well as the M06-2X DFT method recommended for thermochemistry calculations, and compare the discovered results with experimental ones. Investigation of thermochemistry of individual steps of lactone ring opening and successive decarboxylation mechanism, including the identification of transition states, using the M06-2X/6-311++G(d,p) approach, represents another aim of this work.

## 2. Results and Discussion

### 2.1. Enthalpies of Formation in the Liquid Phase

The typical values of combustion experiments of each lactone studied are shown in Table 1. Results of all combustion experiments of each compound are given in the Supplementary Information (Tables S1 and S2).

The standard state correction, known as Washburn correction, Δ*U*_Σ_, is determined as recommended in the literature for organic compounds [8]. The internal energy for the isothermal bomb process, Δ*U*(IBP), is calculated using the Equation (1), where *ε*_cal_ is the energy equivalent of the calorimeter, *ε*_f_ is the energy equivalent of the content in the final state, ∆*T*_ad_ is the corrected temperature rise obtained in each experiment, and ∆*U*(ign) is the electrical energy of ignition. The remaining terms have been previously described [8].

Δ*U*(IBP) = −(*ε*_cal_ + *ε*_f_) ∆*T*_ad_ + ∆*U*(ign), (1)

γUL and δUL being structural isomers, have the same molecular formula. Thus, the respective standard massic energy of combustion is referred to in the combustion reaction described by the chemical Equation (2).

C_11_H_20_O_2_(l) + 15O_2_(g) → 11CO_2_(g) + 10H_2_O(l), (2)

The individual values of standard massic energies of combustion, Δ_c_*u*°, of each compound studied, as well as the mean values with the corresponding standard deviation of the mean, are collected in the Table 2.

The results of standard molar internal energies of combustion, ${∆}_{c}U_{m}^{{}^{\circ}}\left(l\right)$, enthalpy of combustion, ${∆}_{c}H_{m}^{{}^{\circ}}\left(l\right)$, and standard molar enthalpy of formation, ${∆}_{f}H_{m}^{{}^{\circ}}\left(l\right)$, in the liquid phase, at *T* = 298.15 K, are reported in the Table 3. The uncertainties associated with the standard molar internal energy and enthalpy of combustion are twice the overall standard deviation of the mean and include the uncertainties in the calibration with benzoic acid, and in the auxiliary materials used in the combustion experiments [9,10]. To calculate ${∆}_{f}H_{m}^{{}^{\circ}}\left(l\right)$ from ${∆}_{c}H_{m}^{{}^{\circ}}\left(l\right)$, the standard molar enthalpies of formation, at *T* = 298.15 K, were used for H_2_O(l), −(285.830 ± 0.040) kJ·mol^–1^ [11], and CO_2_(g), −(393.51 ± 0.13) kJ·mol^–1^ [11].

### 2.2. Enthalpies of Vaporization

For each compound studied, six experiments were carried out in the Calvet microcalorimeter at *T*~365 K, and the final results are shown in Table 4.

The standard molar enthalpy of vaporization, ${∆}_{l}^{g}{H\;}_{m}^{{}^{\circ}}$, of each lactone was calculated from Equation (3), where the term, ${∆}_{l,\;298.15\;K}^{g,\;T}H_{m}^{\;{}^{\circ}}$, corresponds to the observed enthalpy of vaporization at the experimental temperature (*T*~365 K), and the term ${∆}_{298.15\;K}^{T}H_{m}^{\;{}^{\circ}}\left(g\right)$ corresponds to the correction of the experimental temperature to *T* = 298.15 K. The last parameter (Equation (4)) is obtained using the gas phase molar heat capacities, $C_{p,m}^{{}^{\circ}}(g)$, derived from statistical thermodynamics and the vibrational frequencies obtained at the B3LYP/6-31G(d) level [12], scaled by a factor of 0.9613 [13].

(3) $${∆}_{l}^{g}H_{m}^{{}^{\circ}}={∆}_{l,\;298.15\;K}^{g,\;T}H_{m}^{{}^{\circ}}{-\;∆}_{298.15\;K}^{T}H_{m}^{{}^{\circ}}\left(g\right),$$

(4) $${∆}_{298.15\;K}^{T}H_{m}^{{}^{\circ}}\left(g\right)=\int_{298.15\;K}^{T}C_{p,m}^{{}^{\circ}}\left(g\right)\;dT,$$

The values of $C_{p,m}^{{}^{\circ}}\left(g\right)=f(T)$ are well fitted to third-order polynomials, which are represented in the Equations (5) and (6), for γ-undecanolactone (γUL) and δ-undecanolactone (δUL), respectively. More details about each value of standard molar heat capacity of the lactones studied are given in the Supplementary Information (Table S3).

(5) $$C_{p,m}^{{}^{\circ}}\left(\gamma UL,\;g\right)/\left({J\cdot K}^{-1}{\mathrm{mol}}^{-1}\right)={-1.875\times 10}^{-6}T^{3}+{\;1.970\times 10}^{-3}T^{2}+{5.377\times 10}^{-2}T\;+{\;9.780\times 10}^{1}$$

(6) $$C_{p,m}^{{}^{\circ}}\left(\delta UL,\;g\right)/\left({J\cdot K}^{-1}{\mathrm{mol}}^{-1}\right)={-1.805\times 10}^{-6}T^{3\;}+{1.871\times 10}^{-3}T^{2}+{1.021\times 10}^{-1}T\;+{\;9.081\times 10}^{1}$$

### 2.3. Experimental Enthalpies of Formation in the Gas Phase

The standard (*p*° = 0.1 MPa) molar enthalpies of formation, at *T* = 298.15 K, in the gas phase, ${∆}_{f}H_{m}^{{}^{\circ}}\left(g\right)$, of the lactones were calculated by combining the standard molar enthalpies of formation in the liquid phase and the standard molar enthalpies of vaporization; the results are summarized in the Table 5.

The enthalpic increment for the presence of a methylene group was calculated for the system γ-pentanolactone/γ-hexanolactone [14], −24.4 kJ·mol^–1^. Thus, using the ${∆}_{f}H_{m}^{{}^{\circ}}\left(g\right)$ value of γ-nonanolactone and adding the energetic contribution of two methylene groups, it is possible to estimate the ${∆}_{f}H_{m}^{{}^{\circ}}\left(g\right)$ of the γ-undecanolactone, −538.9 kJ·mol^–1^, which is in agreement with the experimental results obtained in this work. The ${∆}_{f}H_{m}^{{}^{\circ}}\left(g\right)$ value of δ-undecanolactone was also estimated, −535.4 kJ·mol^–1^, summing the energetic contribution of two methylene groups (−24.4 kJ·mol^–1^) with the ${∆}_{f}H_{m}^{{}^{\circ}}\left(g\right)$ value of δ-nonanolactone [15]. These results give us confidence concerning the reliability of the experimental results obtained.

In parallel to the experimental study, the standard molar enthalpy of formation in the gas phase was calculated using quantum chemical calculations.

### 2.4. Enthalpies of Formation in the Gas Phase Derived from Theoretical Calculations

**Figure 2:**
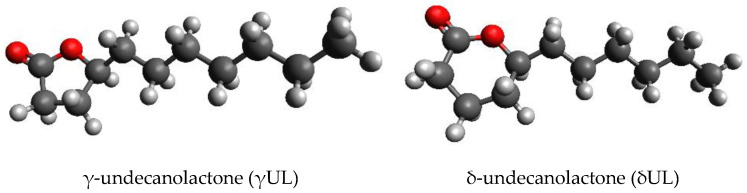
The molecular structures of the most stable conformation of γUL and δUL in the gaseous phase, optimized by the M06-2X/6-311++G(d,p) level of theory. Visualizations were performed in Avogadro program [20].

The investigation of the two lactones started with the identification of the lowest energy conformations. First, the optimum gas-phase geometries of the two lactone rings were optimized using the M06-2X/6-311++G(d,p) approach (Figure 2). Then, the geometry of the molecule with the CH_3_ group instead of the side chain was optimized. The rotational barrier of the attached CH_3_ group was computed as well. The found value of ca. 15 kJ·mol^–1^ is in accordance with the published barrier of the CH_3_ group in the methoxy group of anisole [16,17]. For the CH_2_–CH_3_ side chain, we also performed a 2D scan when both, the CH_2_ and CH_3_ fragments, were rotated, to find the lowest energy conformation. For each additional CH_2_ fragment, the rotational barrier remains ca. 15 kJ·mol^–1^. For species involved in working reactions, corresponding conformations were retained. Thus, the calculated reaction enthalpies and enthalpies of formation are not biased with the effect of conformational changes. From M06-2X/6-311++G(d,p), G4 or G4(MP2) total enthalpies, the reaction enthalpies of individual working reactions were found (Table 6 and Table 7). Using the available enthalpies of formation (Table S4) for the compounds in the working reactions, the enthalpies of formation for the lactone were obtained. From five values, the average, and its uncertainty, *u*_95%_, for the 95% confidence interval were found. In comparison to the experimentally determined enthalpies of formation, it can be concluded that the gas-phased M06-2X/6-311++G(d,p) enthalpies of formation of the two lactones are less negative than experimental ones. However, considering the uncertainties of theoretical and experimental values, M06-2X functional offers results in fair agreement with experiment. Although the composite ab initio G4 method gives results in excellent agreement with the experiment, the computational costs of the G4 calculations are ca. six times higher than M06-2X/6-311++G(d,p). Therefore, the G4(MP2) approach, which uses an approximation based on second-order perturbation theory to lower computational costs [18], may represent a rational choice for thermochemical calculations. With costs analogous to the M06-2X/6-311++G(d,p) method, the discovered results can be considered practically identical to the G4 ones. Although higher errors were reported [19] when the G4(MP2) method was applied to larger organic molecules, for the studied lactones, it provides reliable enthalpies of formation.

For the estimation of the enthalpy of formation of γUL and δUL in the gas phase, a set of five working reactions, (7) to (11) and (12) to (16), respectively, were selected considering the experimental data available in the literature. The computational results at the M06-2X, G4 or G4(MP2) levels are summarized in Table 6 and Table 7. In general, the M06-2X method gives less negative values in comparison to the experimentally determined enthalpies of formation with deviations of 8–12 kJ·mol^–1^. In the case of γUL, Reaction (8) is the one with a result closest to the experimental value, with small deviations of 1.9 kJ·mol^–1^ and –1.4 kJ·mol^–1^, for the G4 and G4(MP2) methods, respectively. The other reactions give results less negative than the discovered experimental value. For δUL, the best agreement between experimental and computed enthalpy of formation shows Reaction (15) with deviations of 2.6 kJ·mol^–1^ for G4 method and 2.9 kJ·mol^–1^ for G4(MP2). The worst agreement shows Reaction (14), with roughly 10 kJ·mol^–1^ deviations for both methods. The results in Table 6 and Table 7 also indicate that the M06-2X functional may represent a rational choice for calculations of larger organic molecules.

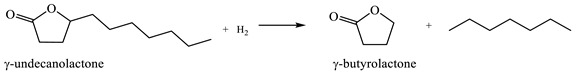
(7)

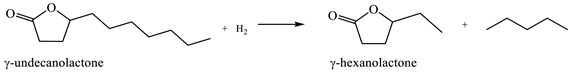
(8)

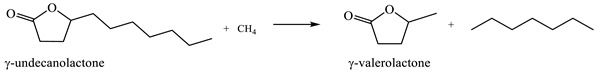
(9)

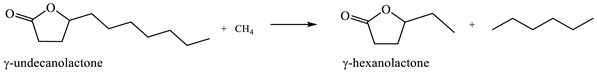
(10)

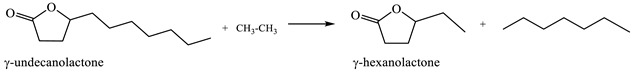
(11)

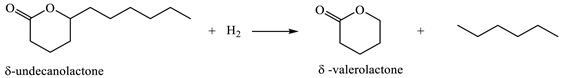
(12)

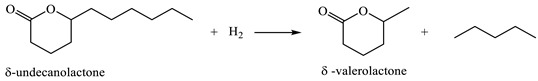
(13)

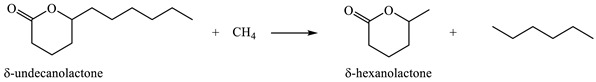
(14)

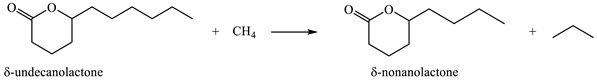
(15)

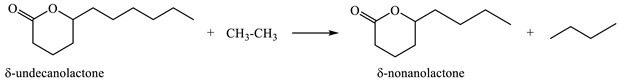
(16)


### 2.5. Estimates of Formation and Vaporization Enthalpies of γ- and δ-Lactones

**Figure 3:**
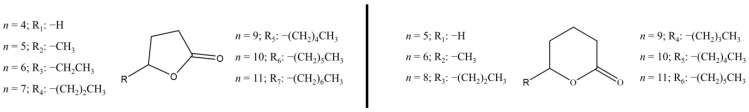
Structures of γ-lactones (left side) and δ-lactones (right side) used in the estimates.

In this work, the estimation of enthalpies of formation in liquid and gaseous phases, as well as the enthalpies of vaporization of γ- and δ-undecanolactones, was carried out. For this purpose, empirical correlation models were obtained by fitting the experimental literature values available for each set of lactones [14,15], as well as the experimental results obtained in this work. Figure 3 presented the structures of lactones used in these correlations and the data are collected in Table 8 and Table 9 for γ- and δ-lactones, respectively.

**Figure 4:**
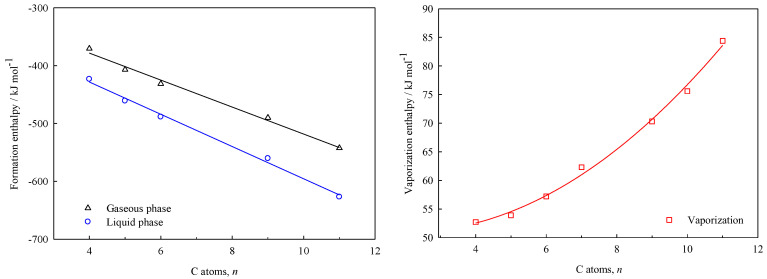
Plots of the standard molar enthalpies of γ-lactones with the respective number of carbon atoms in each structure (Table 8).

**Figure 5:**
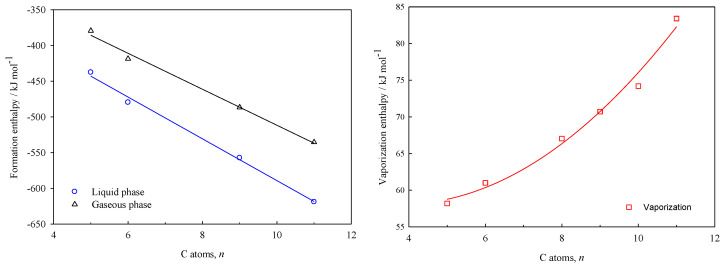
Plots of the standard molar enthalpies of δ-lactones with the respective number of carbon atoms in each structure (Table 9).

The plots of the enthalpies of formation in liquid and gaseous phases, as well as the enthalpies of vaporization vs. the number of carbon atoms (*n*) of each molecule, are shown in Figure 4 and Figure 5, for γ-lactones and δ-lactones, respectively.

The equations derived from the linear and non-linear fits depicted above, are shown in the Equations (17) to (19) for the γ-lactones, and the Equations (20) to (22) for the δ-lactones. This enables the estimation of the energetic properties (${{∆}_{f}H}_{m}^{{}^{\circ}}\left(l\right)$, ${∆}_{l}^{g}H_{m}^{{}^{\circ}}$, ${{∆}_{f}H}_{m}^{{}^{\circ}}\left(g\right)$) for others hydrocarbon-chain lactones that are not experimentally feasible to study, with a standard error of estimates maximum of 8 kJ·mol^–1^.

γ-lactones

(17) $${{∆}_{f}H}_{m}^{{}^{\circ}}\left(l\right)/\left(\mathrm{kJ}\cdot {\mathrm{mol}}^{-1}\right)=-316.2(\pm 8.5)-27.9\;(\pm 1.1)n$$

(18) $${∆}_{l}^{g}H_{m}^{{}^{\circ}}/\left(\mathrm{kJ}\cdot {\mathrm{mol}}^{-1}\right)=52.6(\pm 4.6)-1.6(\pm 1.3)\;n+0.4(\pm 0.1)\;n^{2}$$

(19) $${{∆}_{f}H}_{m}^{{}^{\circ}}\left(g\right)/\left(\mathrm{kJ}\cdot {\mathrm{mol}}^{-1}\right)=-285.1(\pm 9.2)-23.3(\pm 1.2)n$$

δ-lactones

(20) $${{∆}_{f}H}_{m}^{{}^{\circ}}\left(l\right)/\left(\mathrm{kJ}\cdot {\mathrm{mol}}^{-1}\right)=-296.8(\pm 11.4)-29.2\left(\pm 1.4\right)n$$

(21) $${∆}_{l}^{g}H_{m}^{{}^{\circ}}\;/\left(\mathrm{kJ}\cdot {\mathrm{mol}}^{-1}\right)=64.7(\pm 9.8)-3.5(\pm 2.6)\;n+0.5(\pm 0.2)\;n^{2}$$

(22) $${{∆}_{f}H}_{m}^{{}^{\circ}}\left(g\right)/\left(\mathrm{kJ}\cdot {\mathrm{mol}}^{-1}\right)=-260.0(\pm 12.1)-25.2(\pm 1.5)n$$

Finally, in Table 10, the estimated values of the standard molar enthalpies of formation in the liquid and gas phase of lactones with seven, eight and ten carbon atoms in their molecular structure are reported. Additionally, it includes the estimated enthalpy of vaporization values for the eight-carbon γ-lactone and the seven-carbon δ-lactone.

### 2.6. Ring-Opening Hydrogenation of Biomass-Derived Cyclic Oxygenates

**Figure 6:**
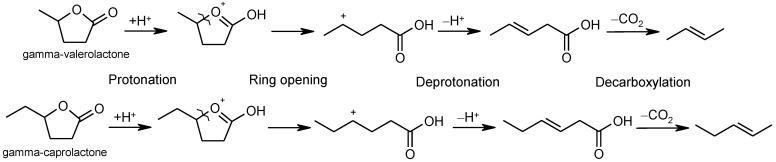
Reaction scheme of final products formation.

Two five-membered ring lactones, i.e., γ-valerolactone and γ-caprolactone containing a methyl and ethyl group instead of a long alkyl side chain, were selected for the investigation of lactone ring opening and successive decarboxylation thermochemistry. These calculations were performed using the (SMD) M06-2X/6-311++G(d,p) and (SMD) G4 methods for aqueous solution. In general, the overall reaction can be formally divided into several steps: (1) protonation of carbonyl group O-atom; (2) ring opening, i.e., C–O bond cleavage; (3) deprotonation of the formed cation; and (4) decarboxylation (Figure 6). The reaction Gibbs free energies of individual steps are summarized in Table 11. We have also calculated the Gibbs free energies of ring O-atom protonation. However, the found results indicate that this process is considerably endergonic and cannot represent the thermodynamically preferred pathway. For the two lactones, the M06-2X method gives a Gibbs free energy of ring O-atom protonation ca. 80 kJ·mol^–1^, and G4 provided ca. 50 kJ·mol^–1^. For the overall reaction, the M06-2X reaction Gibbs free energy predicts a slightly endergonic character, while the G4 data indicate practically zero reaction Gibbs free energy. Data in Table 11 indicate that the length of side chain plays a negligible role in the thermochemistry of the studied reaction. For γ-valerolactone, the experimentally determined standard reaction enthalpy (44.7 ± 4.3 kJ·mol^–1^) and the reaction entropy (71.9 ± 9.1 J·mol^–1^·K^–1^) of lactone ring opening and the formation of pentenoic acid [24] give reaction Gibbs free energy of 23 kJ·mol^–1^ at 298.15 K. With respect to the uncertainty of experimental standard reaction enthalpy, this estimate is in fair agreement with the calculated reaction G4 and M06-2X Gibbs free energies of 29 kJ·mol^–1^ and 31 kJ·mol^–1^ (representing the sum of reaction Gibbs free energies of first three steps), respectively.

**Figure 7:**
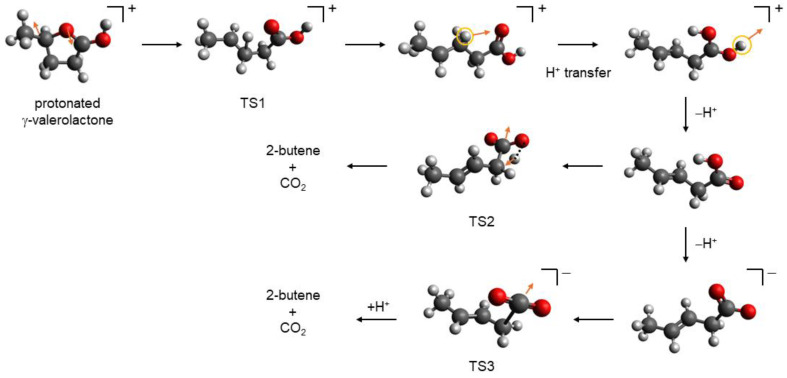
Plausible mechanism of lactone ring opening and successive decarboxylation.

We have also performed calculations to identify the transition states and to find the plausible reaction pathway leading to the formation of final products, i.e., alkene and CO_2_. Due to the geometry optimizations in the small basis set, composite ab initio methods are not appropriate for the calculations of potential energy surfaces and transition states. Therefore, M06-2X calculations were performed. The discovered reaction pathway is identical for both lactones. In Figure 7, the individual steps of the plausible mechanism are shown for γ-valerolactone.

After the protonation of carbonyl group O-atom, ring opening, i.e., C–O bond cleavage occurs. The ring O-atom moves in the opposite direction to the C-atom bearing the methyl group. This movement is caused by the change in dihedral angle defined by four ring C-atoms. For the ring-opening process, the transition state (TS1 in Figure 7), with the barrier of ∆*G*^≠^ = 93 kJ·mol^–1^, was identified. For γ-caprolactone, this barrier is lower by ca. 2 kJ·mol^–1^. The ring-opening process is endergonic with the M06-2X reaction Gibbs free energies of 83 and 85 kJ·mol^–1^ (Table 11) for γ-valerolactone and γ-caprolactone, respectively. After the ring opening, the formed cation undergoes H^+^ transfer to O-atom (their distance is ca. 2.5 Å). This step is exergonic (–40 kJ·mol^–1^) and results in the structure depicted in the right upper corner in Figure 7. For both lactones, this H^+^ transfer requires relatively low activation Gibbs energy of 23 kJ·mol^–1^. Then, exergonic (–34 kJ·mol^–1^) deprotonation from the second O-atom occurs. Successive decarboxylation involves H atom transfer and CO_2_ loss, see the arrows in TS2 in Figure 7. Corresponding reaction Gibbs free energy reached the value of –24 kJ·mol^–1^. The activation Gibbs free energy of 279 kJ·mol^–1^ obtained for the formation of TS2 transition state is identical for the two lactones. The high activation Gibbs free energy explains why the decarboxylation after the lactone ring opening proceeds as a catalyzed reaction at elevated temperatures [24,25,26]. The remaining results obtained for γ-caprolactone can be considered analogous to those for γ-valerolactone. Proton transfer from the C-atom to the neighboring O-atom and consecutive deprotonation are both exergonic with reaction Gibbs free energies of –43 kJ·mol^–1^ and –30 kJ·mol^–1^, respectively. The last step, i.e., the H atom transfer and CO_2_ abstraction, is slightly more exergonic with ∆_r_*G*^o^ = –27 kJ·mol^–1^ in comparison to γ-valerolactone.

Because carboxylic acids in aqueous solutions can be deprotonated, we have also considered the abstraction of the CO_2_ molecule from the corresponding carboxylate anions with successive protonation of formed carbanions. The activation Gibbs free energy value related to corresponding transition state, TS3 in Figure 7, is significantly lower than the value found for TS2. In the case of γ-valerolactone, its value reached 217 kJ·mol^–1^. Although the formation of the carboxylate anion (77 kJ·mol^–1^) and the abstraction of CO_2_ molecule (200 kJ·mol^–1^) are endergonic, the large negative reaction Gibbs free energy of formed carbanion protonation (–301 kJ·mol^–1^) means that the overall process is exergonic with the reaction Gibbs free energy of –24 kJ·mol^–1^. Almost identical results were obtained for γ-caprolactone: the activation Gibbs free energy of TS3 formation reached the value of 215 kJ·mol^–1^; the reaction Gibbs free energies of carboxylate anion formation, CO_2_ abstraction, and carbanion protonation resulting in 2-pentene formation are 75 kJ·mol^–1^, 196 kJ·mol^–1^, and –298 kJ·mol^–1^, respectively.

We should also note that protonation of C3 carbon atom, instead of the C1 atom of carbanion, may lead to the formation of 1-butene from γ-valerolactone or 1-pentene from γ-caprolactone. This pathway is not thermodynamically favored, however; these protonations are only less exergonic by 12 kJ·mol^–1^.

In general, the computational data for the two studied model lactones clearly indicate that the energetics of the investigated processes can be considered practically identical and both reaction and activation. The Gibbs free energies may not depend on the length of the side chain. In general, the mechanism in Figure 7 is compatible with the one proposed in Bond et al. [24,26].

## 3. Materials and Methods

### 3.1. Materials

The samples of γUL (>99% from Sigma-Aldrich, Saint Louis, MO, USA) and δUL (>98% from Sigma-Aldrich) were purified by fractional distillation under reduced pressure and their purity was evaluated by GC equipped with a flame ionization detector. The sample of γUL has been purified by successive fractional distillations, although the maximum degree of purity obtained has been 0.988. The high purity of the compound was confirmed by the consistent results for combustion experiments and, in particular, by the carbon dioxide recovery ratios (average ratio of the mass of the recovered carbon dioxide to that calculated from the mass of sample: 1.0003 ± 0.0003).

A summary of purity control is shown in Table 12. Gas chromatography analysis was carried out on an Agilent (Santa Clara, CA, USA) 4890D gas-chromatography-flame ionization detector apparatus, with a HP-5 column (cross-linked 5% diphenyl and 95% dimethylpolysiloxane; 15 m × 0.530 mm i.d. with 1.5 μm film thickness); nitrogen was used as the carrier gas.

### 3.2. Combustion Calorimetry

The experimental determination of standard (*p*° = 0.1 MPa) massic energy of combustion of each compound was carried out in a static-bomb calorimeter, using a twin valve bomb (internal volume is of 0.342 dm^3^) type 1108 of Parr Instruments Company, described in the literature [27,28].

The energy equivalent of the calorimeter, *ε*_cal_ = (16,002.6 ± 1.7) J·K^–1^, was obtained and corresponds to the average mass of the water added to the calorimeter (3119.6 g). The uncertainty quoted is the standard deviation of the mean of six calibration experiments. This procedure was performed by combustion of the benzoic acid (NIST SRM 39j) with a massic energy of combustion, under bomb conditions, of –(26,434 ± 3) J·g^–1^. The experiments were performed in an oxygen atmosphere at *p* = 3.04 MPa, with 1.00 cm^3^ of deionized water added to the bomb. The calorimeter temperatures were measured to ±0.1 mK, at time intervals of 10 s, using a quartz thermometer (HP-2804), and the initial temperature was as close as possible to 298.15 K.

For each compound, the standard massic energy of combustion, ${∆}_{c}u{}^{\circ}$, was calculated by a similar procedure to that developed by Hubbard et al. [8]. The samples were burnt while enclosed in Melinex^®^ bags (DuPont Teijin Films, Hopewell, VA, USA), which were made according to the technique described by Skinner et al. [29]; the massic energy of combustion of dry Melinex^®^ and respective expanded uncertainty was –(22,902 ± 5) J·g^–1^. Further details about the experimental procedure are provided in Supplementary Information (Section S1).

### 3.3. Calvet Drop Microcalorimetry

The standard molar enthalpies of vaporization of the studied lactones were measured with a high-temperature Calvet microcalorimeter (Setaram HT 1000), using the “vacuum sublimation” drop method described by Skinner et al. [30]. This method was adapted to study of liquid compounds by Ribeiro da Silva [31]. The details of the equipment as well as the technique were previously reported in the literature [32].

In each experiment, the samples of 5 to 8 mg of the compounds, contained in glass capillary tubes, were simultaneously dropped at room temperature (~298.15 K) with the corresponding blank tube, into each microcalorimeter cell. The temperature of the reaction oven of the microcalorimeter was chosen according to the volatility of compounds. In this case, the experimental temperature used for vaporization of both lactones was *T* = 365.5 K. The calibration of microcalorimeter was carried out by the vaporization of undecane at the same temperature, obtaining an internal calibration constant equal to *k* = (1.058 ± 0.002) for γUL and *k* = (1.052 ± 0.001) for δUL.

The standard uncertainty corresponds to the combined standard uncertainty value, which includes the standard deviation of the mean and the standard uncertainty associated with the recommended value of enthalpy of vaporization of reference compound.

### 3.4. Computational Details

Density Functional Theory (DFT) calculations were performed using the Gaussian 16 program package [33]. The optimum gas phase geometries of all species involved in hypothetical working reactions were optimized using hybrid M06-2X [34] functional without any constraints (energy cut-off of 10^−5^ kJ·mol^–1^, final RMS energy gradient under 0.01 kJ·mol^–1^·Å^−1^). Application of sufficiently large 6-311++G(d,p) basis set [35,36] allows a reliable description of the investigated species. The optimum structures were confirmed to be real minima by vibrational analysis. Moreover, the composite ab initio G4 method [37] in the Gaussian 09 program package [38] was also employed for the calculation total enthalpies *H*(*i*). To obtain the reaction enthalpies, at *T* = 298.15 K, Equation (23) was used

(23) $${∆}_{r}H^{{}^{\circ}}=\sum_{i}\nu_{i}H(i),$$

where *ν_i_* is the stoichiometric number of *i*-th reactant or product in the corresponding working reaction. Application of standard gas phase enthalpies of formation, ${\;\Delta }_{f}H^{{}^{\circ}}(i)$, of the remaining compounds, allows us to calculate the enthalpy of formation values for the studied lactones from Equation (24).

(24) $${∆}_{r}H^{{}^{\circ}}=\sum_{i}\nu_{i}{\;\Delta }_{f}H^{{}^{\circ}}(i),$$

Since the G4 method requires considerably high computational costs, we have also employed the G4(MP2) method [18] to compare the results and the reliability of the two methods for studied thermochemistry. Although the G4(MP2) method allows calculations that are 6–8 times faster, increased errors have been found when it is applied to larger organic molecules with 10 or more non-hydrogen atoms [19].

The thermochemistry of the individual steps of the lactone ring opening and successive decarboxylation mechanism, including the identification of transition states, was studied using the M06-2X/6-311++G(d,p) approach [34] because the M06-2X method was optimized for thermochemistry and kinetics calculations of organic compounds reactions.

## 4. Conclusions

The thermochemical experimental study of two long hydrocarbon-chain lactones was carried out using calorimetric techniques. The standard molar enthalpies of formation in the gas phase, and the liquid phase, and the enthalpies of vaporization of the γ- the δ-undecanolactones, are reported. In parallel, computational calculations at the M06-2X, G4 and G4(MP2) level of theory were performed, and the standard molar enthalpies of formation in the gas phase of the studied compounds show very good agreement with the experimental values.

Based on the experimental results obtained and considering the thermodynamic data available in the literature database, we were able to establish linear correlations between the number of carbon atoms and the standard molar enthalpies of formation in liquid and gas phases, as well as vaporization, of several γ- and δ-lactones. Therefore, an alternative approach to experimental and computational analyses for calculating those properties is suggested in this research work.

We have also performed theoretical investigation of lactone ring opening and consecutive decarboxylation of γ-valerolactone and γ-caprolactone at the molecular level to explore the thermochemistry of individual reaction steps and to estimate the reaction barriers for ring opening and decarboxylation steps. These findings further extend insights into the plausible mechanism suggested previously.

Finally, the obtained experimental and theoretical results may find applicability in the research field of green chemistry, because the studied compounds may represent organic solvents and show potential for biofuel applications.

## Figures and Tables

**Table 1:** Typical values of combustion experiments at *T* = 298.15 K and *p*° = 0.1 MPa.

	γUL	δUL
*m*(CO_2_, total)/g	1.24371	1.24077
*m*(cpd)/g *^a^*	0.42148	0.43030
*m*(fuse)/g *^a^*	0.00276	0.00262
*m*(melinex)/g *^a^*	0.05763	0.04632
*T*_i_/K	298.1511	298.1515
*T*_f_/K	299.2529	299.2570
Δ*T*_ad_/K	1.02088	1.02554
*ε*_i_/J·K^–1^	16.54	16.54
ε_f_/J·K^–1^	17.54	17.55
−Δ*U*(IBP)/J	16,353.63	16,428.65
Δ*U*(HNO_3_)/J	1.41	1.51
Δ*U*(ign)/J	1.00	0.65
Δ*U*_Σ_/J	6.62	6.52
−Δ*U*(melinex)/J	1319.74	1060.91
−Δ*U*(fuse)/J	44.82	42.55
−Δ_c_*u*°/(J·g^–1^)	35,543.89	35,596.47

*m*(CO_2_, total) is the mass of CO_2_ recovered in the experiment; *m*(cpd) is the mass of compound burnt in each experiment; *m*(fuse) is the mass of fuse (cotton) used in each experiment; *m*(melinex) is the mass of melinex used in each experiment; *T*_i_ is the initial temperature rise; *T*_f_ is the final temperature rise; ∆*T*_ad_ is the corrected temperature rise; *ε*_i_ and *ε*_f_ are the energy equivalents of contents in the initial and final state, respectively; ∆*U*(IBP) is the energy change for the isothermal combustion reaction under actual bomb conditions; ∆*U*(HNO_3_) is the energy correction for the nitric acid formation; ∆*U*(ign) is the electrical energy for ignition; ∆*U*_Σ_ is the standard state correction; ∆*U*(melinex) is the correction energy for melinex; ∆*U*(fuse) is the energy of combustion of the fuse (cotton); Δ_c_*u*° is the standard massic energy of combustion. *^a^* All masses were adjusted for buoyancy.

**Table 2:** Individual values of standard (*p*° = 0.1 MPa) massic energy of combustion of compounds studied, at *T* = 298.15 K.

γUL	δUL
Δ_c_*u*°/(J·g^–1^)	
35,543.89	35,596.47
35,549.05	35,567.87
35,550.64	35,605.57
35,560.78	35,604.20
35,548.22	35,597.40
35,520.50	35,562.81
<Δ_c_*u*°>/(J·g^–1^) *^a^*	
−(35,545.4 ± 5.6)	−(35,589.1 ± 7.7)

*^a^* Mean value and standard deviation of the mean.

**Table 3:** Standard (*p*° = 0.1 MPa) molar energy of combustion, ${∆}_{c}U_{m}^{{}^{\circ}}\left(l\right)$, enthalpy of combustion, ${∆}_{c}H_{m}^{{}^{\circ}}\left(l\right)$, and enthalpy of formation, ${∆}_{f}H_{m}^{{}^{\circ}}\left(l\right)$, for the compounds studied, at *T* = 298.15 K *^a^*.

Compound	${∆}_{c}U_{m}^{{}^{\circ}}\left(l\right)/\;\mathrm{kJ}\cdot {\mathrm{mol}}^{–1}$	${∆}_{c}H_{m}^{{}^{\circ}}\left(l\right)/\;\mathrm{kJ}\cdot {\mathrm{mol}}^{–1}$	${∆}_{f}H_{m}^{{}^{\circ}}\left(l\right)/\;\mathrm{kJ}\cdot {\mathrm{mol}}^{–1}$
γUL	−6550.1 ± 3.0	−6560.1 ± 3.0	−626.9 ± 3.3
δUL	−6558.2 ± 3.6	−6568.1 ± 3.6	−618.8 ± 3.9

*^a^* Uncertainties are twice the overall standard deviation of the mean, and include the contributions from the calibration and from the auxiliary materials used.

**Table 4:** Standard (*p*° = 0.1 MPa) molar enthalpies of vaporization, ${∆}_{l}^{g}H_{m}^{{}^{\circ}}$, at *T* = 298.15 K for the compounds studied determined by Calvet microcalorimetry.

Compound	*T*_exp_/(K)	${∆}_{l,298.15K}^{g,T}H_{m}^{{}^{\circ}}/\mathrm{kJ}\cdot {\mathrm{mol}}^{-1}$	${∆}_{298.15K}^{T}H_{m}^{{}^{\circ}}(g)/\mathrm{kJ}\cdot {\mathrm{mol}}^{-1}$	${∆}_{l}^{g}H_{m}^{{}^{\circ}}/\;\mathrm{kJ}\cdot {\mathrm{mol}}^{-1}$ *^a^*
γUL	365.52 ± 0.03	102.20 ± 0.54	17.79 ± 0.01	84.4 ± 1.9
δUL	365.51 ± 0.02	101.24 ± 0.54	17.84 ± 0.01	83.4 ± 1.9

*^a^* Uncertainties are twice the overall standard deviation of the mean of six experiments and include the uncertainties in calibration.

**Table 5:** Derived standard (*p*° = 0.1 MPa) molar enthalpies of formation, in the gas phase, at *T* = 298.15 K, for the compounds studied.

Compound	${∆}_{f}H_{m}^{{}^{\circ}}\left(l\right)/\;\mathrm{kJ}\cdot {\mathrm{mol}}^{-1}$	${∆}_{l}^{g}H_{m}^{{}^{\circ}}/\;\mathrm{kJ}\cdot {\mathrm{mol}}^{-1}$	${∆}_{f}H_{m}^{{}^{\circ}}\left(g\right)/\;\mathrm{kJ}\cdot {\mathrm{mol}}^{-1}$ *^a^*
γUL	−626.9 ± 3.3	84.4 ± 1.9	−542.5 ± 3.8
δUL	−618.8 ± 3.9	83.4 ± 1.9	−535.4 ± 4.3

*^a^* Uncertainty calculated through the RSS method.

**Table 6:** Reaction enthalpies of working reactions, ${∆}_{r}H_{m}^{{}^{\circ}}$, and enthalpy of formation, ${∆}_{f}H_{m}^{{}^{\circ}}$ values, in the gas phase, for γ-undecanolactone (γUL).

Reaction	${∆}_{r}H_{m}^{{}^{\circ}}/\;\mathrm{kJ}\cdot {\mathrm{mol}}^{-1}$			${∆}_{f}H_{m}^{{}^{\circ}}/\;\mathrm{kJ}\cdot {\mathrm{mol}}^{-1}$		
	M06-2X	G4	G4(MP2)	M06-2X	G4	G4(MP2)
(7)	−20.6	−16.5	−13.8	−533.5	−537.6	−540.3
(8)	−41.8	−37.7	−34.4	−536.5	−540.6	−543.9
(9)	+10.2	+14.0	+13.3	−530.2	−534.0	−533.3
(10)	+9.7	+14.5	+13.9	−533.6	−538.4	−537.8
(11)	−0.5	+1.9	+1.5	−534.7	−537.1	−536.7
Average				−533.7	−537.5	−538.4
*u*_95%_				3.2	3.3	5.5
Experimental				−542.5 ± 3.8		

**Table 7:** Reaction enthalpies of working reactions, ${∆}_{r}H_{m}^{{}^{\circ}}$, and enthalpy of formation, ${∆}_{f}H_{m}^{{}^{\circ}}$ values, in the gas phase, for δ-undecanolactone (δUL).

Reaction	${∆}_{r}H_{m}^{{}^{\circ}}/\;\mathrm{kJ}\cdot {\mathrm{mol}}^{-1}$			${∆}_{f}H_{m}^{{}^{\circ}}/\;\mathrm{kJ}\cdot {\mathrm{mol}}^{-1}$		
	M06-2X	G4	G4(MP2)	M06-2X	G4	G4(MP2)
(12)	−20.2	−16.2	−13.5	−526.3	−530.3	−533.0
(13)	−39.7	−37.1	−34.0	−525.8	−531.5	−531.5
(14)	+11.8	+15.0	+14.3	−522.9	−526.1	−525.4
(15)	+13.4	+15.9	+15.6	−530.3	−532.8	−532.5
(16)	+2.9	+3.6	+3.3	−531.3	−532.0	−531.7
Average				−527.3	−530.5	−530.8
*u*_95%_				4.3	3.3	3.8
Experimental				−535.4 ± 4.3		

**Table 8:** The literature values of the standard (*p*° = 0.1 MPa) molar enthalpies, at *T* = 298.15 K of γ-lactones *^a^*.

Compound	C atoms, *n*	${∆}_{f}H_{m}^{{}^{\circ}}\left(l\right)/\;\mathrm{kJ}\cdot {\mathrm{mol}}^{-1}$	${∆}_{l}^{g}H_{m}^{{}^{\circ}}/\;\mathrm{kJ}\cdot {\mathrm{mol}}^{-1}$	${∆}_{f}H_{m}^{{}^{\circ}}\left(g\right)/\;\mathrm{kJ}\cdot {\mathrm{mol}}^{-1}$
γ-butyrolactone	4	−423.3 ± 1.0 *^b^*	52.7 ± 1.6 *^b^*	−370.6 ± 1.9 *^b^*
γ-pentanolactone	5	−460.9 ± 1.0	53.9 ± 0.2	−407.0 ± 1.0
γ-hexanolactone	6	−488.6 ± 1.4	57.2 ± 0.3	−431.4 ± 1.5
γ-heptanolactone	7	—	62.3 ± 0.3 *^c^*	—
γ-nonanolactone	9	−560.4 ± 1.7	70.3 ± 0.3	−490.1 ± 1.7
γ-decanolactone	10	—	75.6 ± 0.3 *^c^*	—
γ-undecanolactone	11	−626.9 ± 3.3 *^d^*	84.4 ± 1.9 *^d^*	−542.5 ± 3.8 *^d^*

Values obtained in *^a^* reference [14], *^b^* reference [7], *^c^* reference [21] and *^d^* this work.

**Table 9:** The literature values of the standard (*p*° = 0.1 MPa) molar enthalpies, at *T* = 298.15 K of δ-lactones *^a^*.

Compound	C atoms, *n*	${∆}_{f}H_{m}^{{}^{\circ}}\left(l\right)/\;\mathrm{kJ}\cdot {\mathrm{mol}}^{-1}$	${∆}_{l}^{g}H_{m}^{{}^{\circ}}/\;\mathrm{kJ}\cdot {\mathrm{mol}}^{-1}$	${∆}_{f}H_{m}^{{}^{\circ}}\left(g\right)/\;\mathrm{kJ}\cdot {\mathrm{mol}}^{-1}$
δ-pentanolactone	5	−437.6 ± 0.8 *^b^*	58.2 ± 0.3	−379.4 ± 0.9
δ-hexanolactone	6	−479.6 ± 1.6	61.0 ± 0.1	−418.6 ± 1.6
δ-octanolactone	8	—	67.04 ± 0.21 *^c^*	—
δ-nonanolactone	9	−557.3 ± 2.1	70.7 ± 0.4	−486.6 ± 2.1
δ-dδecanolactone	10	—	74.20 ± 0.25 *^c^*	—
δ-undecanolactone	11	−618.8 ± 3.9 *^d^*	83.4 ± 1.9 *^d^*	−535.4 ± 4.3 *^d^*

Values obtained in *^a^* reference [15], *^b^* reference [22], *^c^* reference [23] and *^d^* this work.

**Table 10:** Standard (*p*° = 0.1 MPa) molar enthalpies estimated at *T* = 298.15 K of γ- and δ-lactones.

Compound	C atoms, *n*	${∆}_{f}H_{m}^{{}^{\circ}}\left(l\right)/\;\mathrm{kJ}\cdot {\mathrm{mol}}^{-1}$	${∆}_{l}^{g}H_{m}^{{}^{\circ}}/\;\mathrm{kJ}\cdot {\mathrm{mol}}^{-1}$	${∆}_{f}H_{m}^{{}^{\circ}}\left(g\right)/\;\mathrm{kJ}\cdot {\mathrm{mol}}^{-1}$
γ-lactones				
γ-heptanolactone	7	−512.0	—	−448.3
γ-octanolactone	8	−539.9	65.7	−471.6
γ-decanolactone	10	−595.7	—	−518.3
δ-lactones				
δ-heptanolactone	7	−501.4	63.1	−436.1
δ-octanolactone	8	−530.6	—	−461.3
δ-decanolactone	10	−589.1	—	−511.6

**Table 11:** Reaction Gibbs free energies, ∆*G*^≠^ in kJ·mol^–1^, of ring opening and successive decarboxylation of γ-valerolactone and γ-caprolactone in aqueous solution at 298.15 K.

Reaction Step.	γ-Valerolactone		γ-Caprolactone	
	M06-2X	G4	M06-2X	G4
Protonation	27	–9	28	–10
Ring opening	83	99	85	102
Deprotonation	–79	–61	–80	–63
Decarboxylation	–19	–29	–21	–31
Overall reaction	12	0	12	–2

**Table 12:** Mass fraction purity and density of the compounds studied.

Compound	CAS No.	Purification Method	Final Mass Fraction Purity	$\rho /g\cdot {\mathrm{cm}}^{-3}$ ^*a*^
γUL	104-67-6	Fraction distillations (*T* = 418 K; *p* = 0.4 kPa)	0.9977 *^b^* 1.0003 ± 0.0003 *^c^*	0.949
δUL	710-04-3	Fraction distillation (*T* = 423 K; *p* = 0.4 kPa)	0.9992 *^b^* 1.0002 ± 0.0003 *^c^*	0.969

*^a^* Refer to the Safety Data Sheet provided by the supplier; *^b^* Method of analysis: GC, Gas–liquid chromatography; *^c^* Purity based on the CO_2_ recoveries.

## Data Availability

Data are contained within the article and Supplementary Materials.

## Supplementary Materials

The following supporting information can be downloaded at: https://www.mdpi.com/article/10.3390/molecules30020399/s1. The data of all the combustion calorimetry experiments of lactones (Tables S1 and S2); the corresponding values of standard molar heat capacities in the gaseous phase of lactones (Table S3) and the computational and literature data necessary to perform the theoretical calculations (Table S4). Refs. [39,40,41,42,43] are cited in Supplementary Materials.
