# Supplementary material for: Thermodynamic Properties of γ- and δ-Lactones: Exploring Alkyl Chain Length Effect and Ring-Opening Reactions for Green Chemistry Applications
Source: Molecules. 2025 Jan 18;30(2):399. doi: 10.3390/molecules30020399 (PMC11767462; doi:10.3390/molecules30020399)
Supplement: Supplementary file 1 [file molecules-30-00399-s001.zip › molecules-3410207-supplementary.pdf]

This supplementary information includes:

- The data of all the combustion calorimetry experiments of lactones (Tables S1 and S2).
- The corresponding values of standard molar heat capacities in the gaseous phase of lactones (Table S3).
- The computational and literature data necessary to perform the theoretical calculations (Table S4)

Acronyms used throughout this supplementary data:
γUL for γ-undecanolactone
δUL for δ-undecanolactone

SECTION S1: Combustion Calorimetry

- The samples of lactones placed in Melinex® bags, were ignited in an oxygen atmosphere (p = 3.04 MPa), with 1.00 cm3 of the deionized water inside to the bomb.
- The cotton thread fuse (empirical formula: $CH_{1.686}O_{0.843}$) had a standard massic energy of combustion [S1], $\Delta_c u°$ = –16240 J·g^{-1}.
- The ignition energy was determined from the change in potential difference on discharge of a 1400 μF condenser across a platinum wire.
- Pressure coefficient of specific energy $(\partial u/\partial p)_T$ = –0.2 J·g^{-1}·MPa^{-1} (typical value for organic compounds [S2]).
- The energetic correction for the nitric acid formation, $\Delta U(HNO_3)$ was based on –59. 7 kJ·mol^{-1} [S3] for the molar energy of formation of 0.1 mol·dm^{-3} HNO_3(aq), from N_2(g), O_2(g), and H_2O(l).
- Specific density of the auxiliaries, at T = 298.15 K: ρ = 1.38 g·cm^{-3} for Melinex® [S4] and ρ = 1.50 g·cm^{-3} for cotton fuse [S5].
- The values of the massic heat capacities, at T = 298.15 K, were calculated as 1.953 J·K^{-1}·g^{-1} for both compounds studied, using Kopp's rule [S6].

Table S1 Standard (p^o = 0.1 MPa) mass energy of combustion of liquid δUL, at T = 298.15 K [a]

$m(CO_2$, total)/g	1.24371	—	1.16422	—	1.22246	1.23358
m(cpd)/g	0.42148	0.41874	0.39325	0.41979	0.41759	0.41888
m(fuse)/g	0.00276	0.00273	0.00284	0.00258	0.00266	0.00244
m(melinex)/g	0.05763	0.05167	0.05525	0.05448	0.05289	0.05642
T_i/K	298.1511	298.1511	298.1506	298.1511	298.1508	298.1506
T_f/K	299.2529	299.2392	299.1906	299.2432	299.2363	299.2425
ΔT_{ad}/K	1.02088	1.00637	0.95504	1.01289	1.00547	1.01239
$\varepsilon_i/J\cdot K^{-1}$	16.54	16.53	16.48	16.53	16.53	16.53
$\varepsilon_f/J\cdot K^{-1}$	17.54	17.52	17.42	17.53	17.52	17.53
$-\Delta U$(IBP)/J	16353.63	16121.14	15298.86	16225.72	16106.89	16217.81
$\Delta U(HNO_3)$/J	1.41	1.17	0.93	1.45	1.41	0.89
ΔU(ign)/J	1.00	1.02	0.89	0.90	0.85	0.80
ΔU_Σ/J	6.62	6.46	6.15	6.53	6.47	6.55
$-\Delta U$(melinex)/J	1319.74	1183.36	1265.37	1247.78	1211.23	1292.10
$-\Delta U$(fuse)/J	44.82	44.34	46.12	41.90	43.20	39.63
$-\Delta_c u^o/(J\cdot g^{-1})$	35543.89	35549.05	35550.64	35560.78	35548.22	35520.05

% CO_2 = (100.03 ± 0.03) [b]

$\langle\Delta_c u^o\rangle = -(35545.4 \pm 5.6)\ J\cdot g^{-1}$ [b]

$m(CO_2$, total) is the mass of CO_2 recovered in the experiment; m(cpd) is the mass of compound burnt in each experiment; m(fuse) is the mass of fuse (cotton) used in each experiment; m(melinex) is the mass of melinex used in each experiment; T_i is the initial temperature rise; T_f is the final temperature rise; ΔT_{ad} is the corrected temperature rise; ε_i and ε_f are the energy equivalents of contents in the initial and final state, respetively; ΔU(IBP) is the energy change for the isothermal combustion reaction under actual bomb conditions; $\Delta U(HNO_3)$ is the energy correction for the nitric acid formation; ΔU(ign) is the electrical energy for ignition; ΔU_Σ is the standard state correction; ΔU(melinex) is the correction energy for melinex; ΔU(fuse) is the energy of combustion of the fuse (cotton); $\Delta_c u^o$ is the standard massic energy of combustion.

[a] All masses were adjusted for buoyancy.

[b] Mean value and standard deviation of the mean.

Table S2 Standard (p^o = 0.1 MPa) mass energy of combustion of liquid δUL, at T = 298.15 K [a]

$m(CO_2$, total)/g	1.24077	1.31128	—	—	1.30528	1.21229
m(cpd)/g	0.43030	0.44403	0.38113	0.39472	0.44824	0.41280
m(fuse)/g	0.00262	0.00243	0.00266	0.00259	0.00275	0.00252
m(melinex)/g	0.04632	0.06150	0.05567	0.05308	0.05383	0.05403
T_i/K	298.1515	298.1504	298.1508	298.1513	298.1504	298.1515
T_f/K	299.2570	299.3049	299.1687	299.1914	299.3063	299.2304
ΔT_{ad}/K	1.02554	1.07677	0.92988	0.95629	1.07630	0.99672
$\varepsilon_i/J\cdot K^{-1}$	16.54	16.59	16.46	16.48	16.59	16.52
$\varepsilon_f/J\cdot K^{-1}$	17.55	17.64	17.37	17.42	17.65	17.50
$-\Delta U$(IBP)/J	16428.65	17249.31	14895.89	15318.90	17241.66	15966.58
$\Delta U(HNO_3)$/J	1.51	1.24	1.38	1.45	1.17	1.41
ΔU(ign)/J	0.65	0.79	0.75	0.88	0.92	0.97
ΔU_Σ/J	6.52	7.03	5.99	6.13	6.95	6.42
$-\Delta U$(melinex)/J	1060.91	1408.38	1274.97	1215.57	1232.70	1237.50
$-\Delta U$(fuse)/J	42.55	39.46	43.20	42.06	44.66	40.92
$-\Delta_c u^o/(J\cdot g^{-1})$	35596.47	35567.87	35605.57	35604.20	35597.40	35562.81

% CO_2 = (100.02 ± 0.03) [b]

$\langle\Delta_c u^o\rangle = -(35589.1 \pm 7.7)\ J\cdot g^{-1}$ [b]

$m(CO_2$, total) is the mass of CO_2 recovered in the experiment; m(cpd) is the mass of compound burnt in each experiment; m(fuse) is the mass of fuse (cotton) used in each experiment; m(melinex) is the mass of melinex used in each experiment; T_i is the initial temperature rise; T_f is the final temperature rise; ΔT_{ad} is the corrected temperature rise; ε_i and ε_f are the energy equivalents of contents in the initial and final state, respetively; ΔU(IBP) is the energy change for the isothermal combustion reaction under actual bomb conditions; $\Delta U(HNO_3)$ is the energy correction for the nitric acid formation; ΔU(ign) is the electrical energy for ignition; ΔU_Σ is the standard state correction; ΔU(melinex) is the correction energy for melinex; ΔU(fuse) is the energy of combustion of the fuse (cotton); $\Delta_c u^o$ is the standard massic energy of combustion.

[a] All masses were adjusted for buoyancy.

[b] Mean value and standard deviation of the mean.

Table S3. Standard (p^{o} = 0.1 MPa) molar heat capacities in the gaseous phase for lactones studied.

T/K	$C^{\circ}_{\mathrm{p,m}}(\mathrm{g})/\mathrm{J\cdot K^{-1}\cdot mol^{-1}}$	
	γUL	γUL
150	144.10	142.33
200	172.16	171.55
250	204.58	204.64
298.15	239.26	239.74
300	240.64	241.12
350	278.11	278.90
400	314.93	315.95
450	349.81	350.97
500	382.14	383.39
550	411.82	413.10

Table S4. M06-2X, G4 and G4(MP2) absolute enthalpies, at T = 298.15 K and literature values of the standard molar enthalpies of formation of the species used in this work [a]

Molecules	Absolute enthalpies / Hartree			$\Delta_f H_m^\circ(g)$ / kJ·mol^{-1}
	M06-2X	G4	G4(MP2)	
Dihydrogen	-1.15491	-1.164715	-1.167103	—
γUL	-581.270737	-581.274256	-580.760829	−542.5 ± 3.8[b]
δUL	-581.267706	-581.270402	-580.756941	−535.4 ± 4.3[b]
Methane	-40.447977	-40.461493	-40.423842	-74.4 ± 0.4 [S7]
Ethane	-79.717588	-79.733656	-79.657478	-83.8 ± 0.3 [S7]
Propane	-118.991213	-119.010264	-118.895577	-104.7 ± 0.5 [S7]
Pentane	-197.538232	-197.564002	-197.372199	-146.9 ± 0.8 [S7]
Hexane	-236.811692	-236.840923	-236.610544	-166.9 ± 0.8 [S7]
Heptane	-276.085168	-276.117872	-275.848906	-187.6 ± 1.3 [S7]
γ-Butyrolactone	-306.348332	-306.327381	-306.08429	–366.5 ± 0.8 [S8]
γ-Valerolactone	-345.629659	-345.612539	-345.330686	–406.5 ± 1.1 [S8]<br>–407.0 ± 1.0 [S9]
γ-Hexanolactone	-384.903342	-384.889317	-384.568846	–431.4 ± 1.5 [S9]
δ-Valerolactone	-345.618624	-345.600353	-345.318633	–379.6 ± 0.9 [S8]
δ-Hexanolactone	-384.899509	-384.885264	-384.564787	–418.6 ± 1.6 [S9]
δ-Nonanolactone	-502.719376	-502.715567	-502.279273	–486.6 ± 2.1 [S9]

[a] Atomic unit of energy Hartree: 1 E_h = 2625.50184 kJ·mol^{-1}.
[b] This work.

References

[S1] J. Coops, R.S. Jessup, K. van Nes, Calibration of calorimeters for reactions in a bomb at constant volume, in: F.D. Rossini (Ed), Experimental Thermochemistry. Measurement of Heats of Reaction, Interscience Publishers Inc., New York, 1956, pp. 27-58.

[S2] E.W. Washburn, Standard states for bomb calorimetry, J. Res. Nat. Bur. Stand. 10 (1933) 525-558. https://doi.org/10.6028/jres.010.037.

[S3] D.D. Wagman, W.H. Evans, V.B. Parker, R.H. Schumm, I. Halow, S.M. Bailey, K.L. Churney, R.L. Nutall, The NBS Tables of Chemical Thermodynamics Properties, J. Phys. Chem. Ref. Data II 11 (1982) Suppl. 2. https://doi.org/doi:10.18434/M32124.

[S4] H.A. Skinner, A. Snelson, The heats of combustion of the four isomeric butyl alcohols, Trans. Faraday Soc. 59 (1960) 1776-1783. https://doi.org/10.1039/TF9605601776.

[S5] W.N. Hubbard, D.W. Scott, G. Waddington, Standard states and corrections for combustions in a bomb at constant volume, in: F.D. Rossini (Ed), Experimental Thermochemistry. Measurement of Heats of Reaction, Interscience Publishers Inc., New York, 1956, pp. 75-128.

[S6] J.E. Hurst, B.K. Harrison, Estimation of liquid and solid heat capacities using a modified Kopp's rule, Chem. Eng. Comm. 112 (1992) 21-30. https://doi.org/10.1080/00986449208935989.

[S7] J.P. Pedley, Thermochemical data and structures of organic compounds, Thermodynamics Research Centre, College Station, TX, 1994.

[S8] M.L.P. Leitão, G. Pilcher, Y. Meng-Yan, J.M. Brown, A.D. Conn, Enthalpies of combustion of γ-butyrolactone, γ-valerolactone, and δ-valerolactone, J. Chem. Thermodyn. 22 (1990) 885-891. https://doi.org/10.1016/0021-9614(90)90176-Q.

[S9] V.N. Emel'yanenko, S.P. Verevkin, E. N. Burakova, G. N. Roganov, M.K. Georgieva, The thermodynamic properties of alkylated γ-lactones, Russ. J. Phys. Chem. A 83 (2009) 598-603. https://doi.org/10.1134/S0036024409040141.
